# Can Ketogenic Diet Improve Alzheimer's Disease? Association With Anxiety, Depression, and Glutamate System

**DOI:** 10.3389/fnut.2021.744398

**Published:** 2021-10-27

**Authors:** Jose Enrique de la Rubia Ortí, David Fernández, Félix Platero, María Pilar García-Pardo

**Affiliations:** ^1^Department of Nursing, Catholic University of Valencia, Valencia, Spain; ^2^Department of Medicine, University of Valencia, Valencia, Spain; ^3^Department of Psychology and Sociology, University of Zaragoza, Teruel, Spain

**Keywords:** Alzheimer's disease, ketogenic diets, anxiety, depression, glutamate

## Abstract

**Background:** Alzheimer's disease is the most common neurodegenerative disorder in our society, mainly characterized by loss of cognitive function. However, other symptoms such as anxiety and depression have been described in patients. The process is mediated by alterations in the synaptic and extrasynaptic activity of the neurotransmitter glutamate, which are linked to a hypometabolism of glucose as the main source of brain energy. In that respect, Ketogenic diet (KD) has been proposed as a non-pharmacological treatment serving as an alternative energy source to the neurons increasing the fat percentage and reducing the carbohydrates percentage, showing promising results to improve the cognitive symptoms associated with different neurodegenerative disorders, including AD. However, the association of this type of diet with emotional symptoms and the modulation of glutamate neurotransmission systems after this dietary reduction of carbohydrates are unknown.

**Objective:** The aim of this short review is to provide update studies and discuss about the relationship between KD, anxiety, depression, and glutamate activity in AD patients.

**Discussion:** The main results suggest that the KD is an alternative energy source for neurons in AD with positive consequences for the brain at different levels such as epigenetic, metabolic and signaling, and that the substitution of carbohydrates for fats is also associated with emotional symptoms and glutamate activity in AD.

## Introduction

At present, Alzheimer's disease (AD) is the most prevalent form of dementia, appearing mainly in the elderly and defined by a prematurely aging brain. To date there is no cure, affecting more than 50 million people worldwide (1). This disease is characterized by a progressive and irreversible memory loss. However, related to that aging brain, neuropsychiatric symptoms are also very relevant in Alzheimer's-type disorder; specially, the presence of anxiety and depression (2), which have a direct impact on the quality of life of patients (3). This is why, although they are not usually given as much attention at the therapeutic level, an adequate treatment of these neuropsychiatric symptoms could considerably improve the quality of life, related at the same time to a better prognosis of the disease (4). Anxiety and depression in AD patients are treated pharmacologically. Nonetheless, many problems linked to the use of these drugs have been described in these patients, with even greater progression and development of the disease (5–7), neuronal damage, and, in addition, mature neurons becoming immature, which could explain why antidepressants also induce apoptosis (8).

With this in mind, it is necessary to consider other non-pharmacological options that do not pose risks to patients to improve their symptoms. In this regard, KDs, rich in medium chain fatty acids (MCFA), show promising results. This type of diet is an alternative source of energy to glucose which could improve the different symptoms of AD. By shifting metabolism from carbohydrates toward fatty acids, it has been seen that KD are able to stimulate the production of ketone bodies after hepatic metabolism, which will be used as a new energy option by the central nervous system (9). It seems that a diet based on high-fat and low-carbohydrate content induces the body to a ketosis state similar to the effect of fasting, generating a neuroprotective action on aging brain cells, reducing brain inflammation, and improving mitochondrial function (10). Specifically related to the energy activity in mitochondria, it is known that in neurodegenerative disorders there is a disruption of the brain's energy metabolism, therefore, ketone bodies can support brain energy and slow the progression of different neurodegenerative disorders such as AD (11). In fact, different current studies have evidenced the mechanism of KD for AD treatment and prevention (12–14). This activity promoted by KD intake could consequently justify not only improvements in cognitive disfunctions (15, 16), but also in mood state disorders using 3xTgAD mouse models of AD (17, 18).

Despite the evidence about the positive effect of KD and AD, further research is necessary on the etiopathogenesis of this disease that causes known neurophysiological alterations, in order to understand all the mechanisms through which this type of diet achieves improvements. In this regard, the presence and accumulation of β-amyloid (Aβ) proteins plaques seems to be particularly noteworthy (19), since there is an association between the emotional symptoms and the deposition of Aβ associated with cognitive deterioration (20), and it is even possible to see that the interaction of these variables with the amyloid state can be used to predict the speed of cognitive decline (21). As the origin of this increase in the deposition of the amyloid protein as well as the genetic causes (22, 23), alterations in the levels of neurotransmitters are also highlighted, especially the decrease in the neurotransmitter acetylcholine (24) and the increase in glutamate levels (25). Furthermore, metabolic disorders are also considered important, especially those related to insulin resistance in the brain, which would result in a misuse of glucose by certain regions involved in the development of the disease, even appearing to have a kind of insulin resistance in the brain or “type 3 diabetes” (26). Several studies have remarked the role the apolipoprotein E allele 4 (APOE4) as a common risk factor for AD and type 2 diabetes. Metabolic profiling showed that the APOE4 variant is specifically associated with one type of AD related to decreased brain glucose utilization. In fact, one and two APOE4 alleles have been used as biomarkers of AD, since carriers of this alleles showed decreased brain glucose uptake even years before the onset of clinical symptoms of AD (27–30).

Therefore, although several papers in recent years have reported the role of KD on AD, it seems that the relationship between this both variables (diet and brain disease) is complex and influenced by several factors. As it has been previously mentioned, mood disorders and alterations in different neurotransmitters have been observed in AD patients. However, very few articles have studied whether there is a relationship between the KD, mood state, neurotransmission brain systems, and AD. Thus, the aim of this short review is to provide the most current scientific evidence that shows the association between these variables in order to contribute to new therapeutical strategies for AD.

## Impact of Glutamate on Anxiety and Depression in Alzheimer's Disease (AD)

As for the alteration of the activity of certain neurotransmitters, excess glutamate seems to be directly related to the emotional aspects of dementia like AD, specially outlining the perception of anxiety and depression (31). This is due to the fact that in patients with AD, as a result of excessive levels of this neurotransmitter in the extrasynaptic space, there is hyperexcitability in neurons, with overstimulation especially in NMDA ionotropic receptors, leading to synaptic loss and cell death due to an increase in the cytoplasmic concentration of Ca^++^ and the generation of reactive oxygen species. This process is mainly due to a lack of activity in the glutamate transporters in charge of eliminating excess levels of the neurotransmitter, which is related to the presence of amyloid plaques (32). In this respect, when attempting to explain this relation, two mechanisms tied to different glutamate receptors have always been observed in the hippocampus.

On the one hand, a clear decrease in anxiety levels was observed when postsynaptic group II metabotropic glutamate receptors (mGluRs) were blocked in mice with AD, specifically mGlu2 and mGlu3 (mGluR2/3), using as a model Dutch mice APP (Alzheimer's amyloid precursor protein E693Q), transgenic rodents that accumulate Dutch amyloid-β (Aβ) oligomers. This is due to high levels of glutamate, which have been observed to activate these receptors in the hippocampus, increasing the production of Aβ42 amyloid peptide and not Aβ40 with less aggregation, increasing the proportion of Aβ42:Aβ40 and, in turn, the ability to form amyloid beta plaques, which decrease neurogenesis and promote the onset of anxiety and depression (33). Moreover, Kim et al. demonstrated that APP transgenic mice showed phenotype changes after treatment with BCI-838 (a drug that acts as a Group II mGluR antagonist metabolite) for 3 months. In particular, it was observed that this treatment was associated with reversal of transgene-related amnestic behavior and reduced anxiety levels (33). On the other hand, NMDA ionotropic glutamate receptors are abundantly expressed throughout the whole brain, carrying out an essential function, not only at a cognitive level (34), but also regarding anxiety (35) and depression (36) by having an impact on synaptic plasticity. This could be explained, in part, by the amyloid-β peptide inducing the liberation of astrocytic glutamate (through its cross-interaction with nicotinic acetylcholine receptors and the entry of Ca^2+^ needed for the release of glutamate), which at the same time activates the extrasynaptic NMDA receptors (eNMDAR) in the neurons. The action of these eNMDAR causes an inward current in excess of Ca^2+^, which sequentially stimulates the neuronal nitric oxide synthase (nNOS) generating high levels of NO, which contributes to the loss of synaptic spines (37). Blocking these receptors in animal models [adult male Wistar rats induced with sporadic Alzheimer's-like disease using microinjections of streptozotocin (3 mg/kg/5 μl)], decreased the perception of anxiety and depression. This was associated with a reduction in inflammation mediated by inflammatory cytokines, such as interleukins IL-6 and IL-1β, and tumor necrosis factor alpha (TNF-α) (38). Therefore, regulating glutamate activity in this brain area, will not only achieve cognitive improvements (39), but it is also related to levels of anxiety and depression (40, 41), and there is evidence that glutamate receptors can alter cognitive and mood state both in humans and model animals, using transgenic mouse models that have specific receptor subunits that can be targeted in specific brain regions. However, these studies have limitations since it is difficult to understand where glutamate antagonists act to induce anxiolytic or antidepressant effects and to assess the phenotypes in the animals.

**Figure 1:**
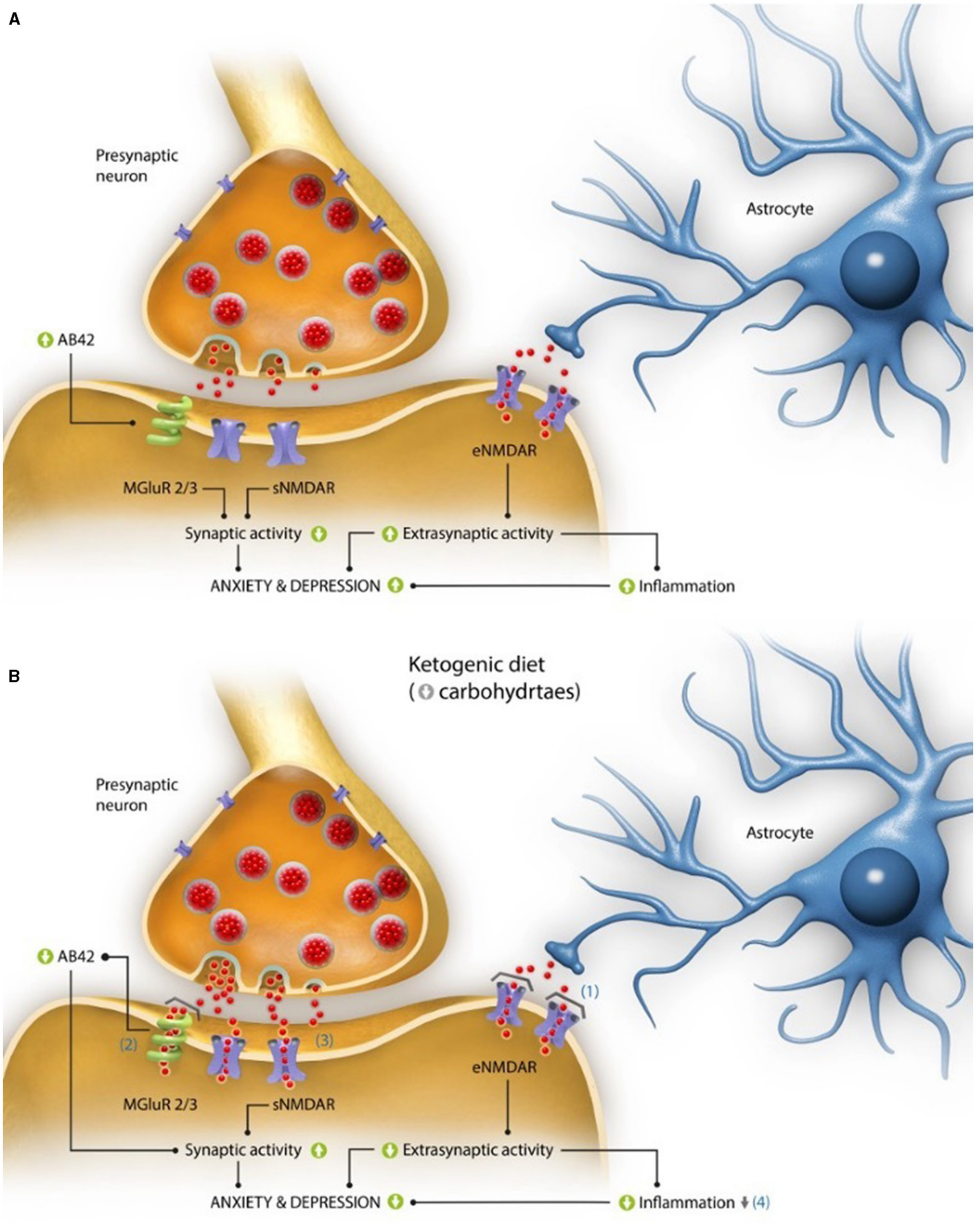
Interaction of glutamate activity on alterations in anxiety and depression levels characteristic of the disease. **(A)** Pathogenic mechanisms based on the activity of the excitatory neurotransmitter glutamate both at synaptic and extrasynaptic levels, which could explain the elevated perception of anxiety and depression described in Alzheimer's disease (AD). At the synaptic level, in the animal model of the disease 3xTgAD mice the activation of mGluR2/3 receptors due to excess glutamate (red dots), has been linked to the formation of β-amyloid peptides with 42 residues long (Aβ42), while at the extrasynaptic level, it has been linked to high glutamate levels, which can increase the activation of its NMDA receptors (eNMDAR), producing an increase in inflammation. Both processes have been linked to the presence of anxiety and depression. **(B)** Proposed mechanisms of action of a ketogenic diet (KD) in the improvement of perception of anxiety and depression in patients with AD. (1) The production of ketone bodies derived from the intake of KDs act as glutamate inhibitors in the NMDA extrasynaptic receptor (eNMDAR), decreasing the extrasynaptic activity of glutamate (red dots) and, as a consequence, the inflammation. (2) They are also capable of blocking the toxicity derived from the formation of amyloid plaques, whose production is partly due to the activation of the mGluR2/3 glutamate receptors. (3) Moreover, they could improve the activity of glutamate at a synaptic level because of a greater ATP contribution (with regard to glucose metabolism), which would have a positive impact on the cognitive and emotional capacity. (4) Finally, the neuroprotector effect of ketone bodies (as a result of the improvement in the electron chain functioning) could lessen the levels of oxidative stress and inflammation. All these processes achieve a decrease in the perception of anxiety and depression, characteristic of this pathology.

Conversely, it should be noted that the glutamate role in its binding to NMDA receptors at a synaptic level (sNMDAR) is also essential for cognition and neuron survival (42, 43), and precisely in AD there is glucose hypometabolism in certain brain areas, possibly linked to an insulin resistance as aforementioned. This hypometabolism implies that the glutamate neurotransmission (GNT) at a synaptic level, which requires a glial-neuronal process with oxidation of glucose and the glutamine-glutamate cycle (44), consuming up to the 80% of ATP provided by the metabolism of glucose (45), may be diminished, and that there is no adequate synapse in its NMDA receptors. As consequence, it can be concluded that in AD the activation of sNMDAR initiates plasticity and stimulates cell survival, while the stimulation of eNMDAR promotes cell death (46). Therefore, these two different groups of glutamate receptors seem to be connected with depression and anxiety (Figure 1A).

## Discussion

As it has been showed throughout this work, the importance of non-pharmacological therapies in AD is essential to improve symptoms and to learn about different novel treatments. Among them, KD has demonstrated great results on the progression of many neurodegenerative disorders (11). Specifically, in AD the data is promising and several studies have evidenced the positive effect of this type of diet in this disorder in both animals and humans (47, 48).

The KD is actually a biochemical model of fasting. Glucose is known to be the main energy source to the neurons. However, in some conditions such as food deprivation or under fasting, brain cells use other alternative energy sources, like ketone bodies. Under these circumstances the human body starts to use fats from its own deposits with a consequent ketosis (49). This type of diet that replaces carbohydrates with fats has positive consequences on the brain at an epigenetic, metabolic and signaling level (50). On the other hand, some recent studies have showed that the neuroprotective effects of KD might be explained by indirect actions on neurons. It has been seen that there are changes in the microbiome after following this type of diet, related to an improvement in the gut-brain axis (51).

As far as the distribution of calories is concerned, in KD, 90% of the total calorie intake is from fat, while only 6% is from protein and 4% from carbohydrates (52). This can be achieved by a composition characterized by a macronutrient ratio of 4:1 (4 g of fat every 1 g of protein and carbohydrates) (50), reducing carbohydrates to ≤ 10% of the energy consumed (53). Nonetheless, there are some alternatives that slightly change the proportion of carbohydrates, such as de Atkins diet in which these are limited to 5% of dietary energy (54) obtaining interesting cognitive improvements (55). Besides, this ketogenesis is more effective when the fats, instead of being long chain fatty acids (LCFA) (which represents the classical version of the diet) (54) are medium chain triglycerides (MCT) made up of MCFA, as it increases the concentration of ketone bodies in blood even if carbohydrates are present in diet, making it a less restrictive diet and easier to follow (56). Precisely both preclinical (57) and clinical studies including a diet enriched with foods high in MCTs, such as coconut oil (58), has showed the positive effect of this diet. These improvements, in particular achieved with MCFA, could also be related to the metabolic activity that has been evidenced in astrocytes, where especially the administration of caprylic acid with 8 C atoms (C8:0) does not affect glycolysis, but clearly increases ketogenesis (59), so that MCFA may have benefits through the modulation of astrocyte metabolism, providing energy to neighboring neurons especially through ketone bodies (60). Moreover, concretely in AD itself, even without an extern MCFA source, it has been shown how the gliosis derived from the disease causes the astrocytes themselves to protect and repair the lesion by optimizing their metabolism through the synthesis of ketone bodies (61).

However, the influence on this type of diet on other variables of a different nature, such as neurotransmission systems or mood variables, has not been studied. In this work it has been described that the mood state and glutamate neurotransmission system can be involved on the effect of KD in AD. Regarding mood state, this work suggests that emotional improvements may be a consequence of a direct action of ketone bodies in relation to extrasynaptic glutamatergic receptors eNMDAR. It has been observed that acetone and β-hydroxybutyrate (βHB) act as glutamate inhibitors in NMDA receptor, specifically highlighting the activity exhibited by βHB, which inhibits the effects of agonists of these receptors at concentrations achieved in *vivo* (62). This process could be related to the observed decrease in glutamate availability in a neuron culture in which glucose is replaced by βHB as an energy source (63). It must be added that the improvements in anxiety and depression observed in AD patients could also be related to the protection ketone bodies seem to exert on cortical neurons against the β-amyloid induced toxicity (64). This mechanism of action could suggest that, even though ketone bodies have not been directly linked to changes in the activity of mGluR receptors, it has been demonstrated in animal models (using male 3xTgAD mice of the disease) that these diets significantly decrease β-amyloid peptide in the brain, which is in turn related in part to the activation of these receptors (18).

Furthermore, ketone bodies acetoacetate and βHB, after crossing the blood-brain barrier, can replace glycolysis. This change would improve glutamate activity at a synaptic level, with a better ATP efficiency; since the metabolites would act as energy substrates of complex II of the respiratory chain, bypassing complex I (which, together with complex IV, are the ones altered in the majority of diseases of mitochondrial nature) (65). Therefore, ketone bodies provide an energy source with higher ATP yield than glucose (66), which may also improve metabolic alterations due to a misuse of glucose, characteristic of the disease caused by destruction of the locus coeruleus (67).

Along these lines, improvements in the functioning of the electron chain in oxidative phosphorylation mediated by ketone bodies achieve cognitive and emotional betterments, given the link established between the mitochondrial alterations and the presence of this symptomatology (68); as a consequence of the decrease in the level of oxidative stress and inflammation, related, in turn, with the presence of anxiety and depression (69, 70).

All these processes are shown in Figure 1B.

In short, due to the negative effects associated to pharmacological treatments for anxiety and depression in AD, the increase of ketone bodies in blood after the administration of KDs (based on the low levels of hydrates and high levels of fat), could be an effective option for the treatment of both, not only for their neuroprotective activity (71, 72) but for their interaction in the pathogenic mechanisms of the disease mediated directly or indirectly by the glutamate activity.

It should also be considered at what point in the disease the KD could be more effective. Studies show how improvement in episodic memory, and reported vitality occurs in patients with mild cognitive impairment (MCI) on early AD after the administration of the modified Atkins diet (MAD) (55). This is in line with results in our laboratory, where the administration of coconut oil rich in MCFA improved episodic orientation and temporal and semantic memory, mainly in the mild-moderate stage of the disease (58). The explanation for these results may be due to the fact that energy hypometabolism begins to occur even decades before the onset of clinical symptoms progressing in the early stages of the disease, as we have previously highlighted (73, 74). However, in the severe phase of the disease, possibly as a consequence of the prolonged bioenergetic deficits and the high oxidative stress derived from these alterations, there is an increase in amyloid plaques (75) that activate apoptotic pathways, aberrant mitochondrial biogenesis and altered mitophagy resulting in neuronal death (76). Thus, the phase of the disease in which the diets are administered should be considered to improve their efficacy. Therefore, it could be therapeutically beneficial in the initial phases to combine diets with other nutraceutical or pharmacological treatments aimed at curbing the high oxidative stress associated with glucidic hypometabolism (77) and, in advanced stages, the combination of KDs should be given with drugs that treat the pathologic signs of the disease, fundamentally related to the formation of amyloid plaques. In this regard, the efficacy of different antioxidants that prevent and reverse AD when combined with adequate diets has been seen (78), highlighting vitamin C (79), α-lipoic acid (80) or the polyphenols epigallocatechin gallate and resveratrol (which can also prevent the neurotoxic effects of β-amyloid protein) (81), while drugs such as donepezil, galantamine and rivastigmine, which act as inhibitors of acetylcholinesterase derived from the accumulation of β-amyloid, could be more effective in advanced stages of the disease (82). Finally, and directly related to anxiety and depression variables, main focus of our study, the combination with glutamate inhibitors such as memantine or lamotrigine could improve the effectiveness of the impact of this diet on these variables, by completing the mechanisms related to the neurotransmitter already analyzed (83–86).

To conclude, and despite the benefits discussed and analyzed in this work, it is important to remark that in some studies in which KD were followed, adverse effects could be observed, mainly focused on gastrointestinal symptoms (constipation, nausea, vomiting and decreased appetite) (87–89), which even forced the interruption of the treatment (56) and transient hyperlipidaemia (90), seeing an increase in fasting serum total cholesterol, triglycerides and low-density lipoprotein (LDL) at the beginning of the treatment (91). In addition, as for the efficacy of KD, a recent review has highlighted the positive cognitive assessments obtained in the short term, and there are no published studies that have conducted follow-ups to determine whether the improvements in variables such as anxiety and depression are maintained over time, or even when the diet is discontinued (92). It should also be noted that these diets usually result in weight loss (93) and in that this loss is common, detrimental and predictive of the cognitive state of Alzheimer's patients (94), so it should be assessed and considered throughout the treatment. Therefore, more studies in this area are needed to gain further knowledge of this disease and the variables involved.

## Data Availability Statement

The original contributions presented in the study are included in the article/supplementary material, further inquiries can be directed to the corresponding author/s.

## Author Contributions

MPG-P and JR: developed the hypothesis and wrote the manuscript. FP and DF: writing-review and editing. All authors contributed to the article and approved the submitted version.

## Funding

The authors were funded by the Catholic University Foundation San Vicente Mártir.

## Conflict of Interest

The authors declare that the research was conducted in the absence of any commercial or financial relationships that could be construed as a potential conflict of interest.

## Publisher's Note

All claims expressed in this article are solely those of the authors and do not necessarily represent those of their affiliated organizations, or those of the publisher, the editors and the reviewers. Any product that may be evaluated in this article, or claim that may be made by its manufacturer, is not guaranteed or endorsed by the publisher.

## References

[1] Soria Lopez JA González HM Léger GC. Alzheimer's disease. Handb Clin Neurol. (2019) 167:231–55. 10.1016/B978-0-12-804766-8.00013-3 31753135

[2] Chen JC Borson S Scanlan JM. Stage-specific prevalence of behavioral symptoms in Alzheimer's disease in a multi-ethnic community sample. Am J Geriatr Psychiatr. (2000) 8:123–33. 10.1097/00019442-200005000-00007 10804073

[3] Shin IS Carter M Masterman D Fairbanks L Cummings JL. Neuropsychiatric symptoms and quality of life in Alzheimer disease. Am J Geriatr Psychiatr. (2005) 13:469–74. 10.1097/00019442-200506000-00005 15956266

[4] Tschanz JAT Corcoran CD Schwartz S Treiber K Green RC Norton MC. Progression of cognitive, functional and neuropsychiatric symptom domains in a population cohort with Alzheimer's dementia: the cache county dementia progression study. Am J Geriatr Psychiatry. (2011) 19:532–42. 10.1097/JGP.0b013e3181faec23 21606896 PMC3101372

[5] Biétry FA Pfeil AM Reich O Schwenkglenks M Meier CR. Benzodiazepine use and risk of developing Alzheimer's disease: a case-control study based on Swiss claims data. CNS Drugs. (2017) 31:245–51. 10.1007/s40263-016-0404-x 28078633

[6] Gray SL Anderson M Hubbard R. Anticholinergic use with incident dementia-reply. JAMA Intern Med. (2015) 175:1577. 10.1001/jamainternmed.2015.2574 26348509

[7] Borda MG Jaramillo-Jimenez A Oesterhus R Santacruz JM Tovar-Rios DA Soennesyn H. Benzodiazepines and antidepressants: effects on cognitive and functional decline in Alzheimer's disease and lewy body dementia. Int J Geriatr Psychiatry. (2021) 36:917–25. 10.1002/gps.5494 33382911

[8] Andrews PW Anderson Thomson J Amstadter A Neale MC. Primum non nocere: an evolutionary analysis of whether antidepressants do more harm than good. Front Psychol. (2012) 3:117. 10.3389/fpsyg.2012.00117 22536191 PMC3334530

[9] Owen OE Morgan AP Kemp HG Sullivan JM Herrera MG Cahill GF. Brain metabolism during fasting. J Clin Invest. (1967) 46:1589–95. 10.1172/JCI105650 6061736 PMC292907

[10] Rusek M Pluta R Ułamek-Kozioł M Czuczwar SJ. KD in Alzheimer's disease. Int J Mol Sci. (2019) 20:3892. 10.3390/ijms20163892 31405021 PMC6720297

[11] Jensen NJ Wodschow HZ Nilsson M Rungby J. Effects of ketone bodies on brain metabolism and function in neurodegenerative diseases. Int J Mol Sci. (2020) 21:8767. 10.3390/ijms21228767 33233502 PMC7699472

[12] Uddin MS Kabir MT Tewari D Al Mamun A Barreto GE Bungau SG. Emerging therapeutic promise of ketogenic diet to attenuate neuropathological alterations in Alzheimer's disease. Mol Neurobiol. (2020) 57:4961–77. 10.1007/s12035-020-02065-3 32820459

[13] Augustin K Khabbush A Williams S Eaton S Orford M Cross JH. Mechanisms of action for the medium-chain triglyceride KD in neurological and metabolic disorders. Lancet Neurol. (2018) 17:84−93. 10.1016/S1474-4422(17)30408-8 29263011

[14] Broom GM Shaw IC Rucklidge JJ. The KD as a potential treatment and prevention strategy for Alzheimer's disease. Nutrition. (2019) 60:118−21. 10.1016/j.nut.2018.10.003 30554068

[15] Ota M Matsuo J Ishida I Hattori K Teraishi T Tonouchi H. Effect of a ketogenic meal on cognitive function in elderly adults: potential for cognitive enhancement. Psychopharmacology. (2016) 233:3797–802. 10.1007/s00213-016-4414-7 27568199

[16] Ota M Matsuo J Ishida I Takano H Yokoi Y Hori H. Effects of a medium-chain triglyceride-based ketogenic formula on cognitive function in patients with mild-to-moderate Alzheimer's disease. Neurosci Lett. (2019) 690:232–6. 10.1016/j.neulet.2018.10.048 30367958

[17] Pawlosky RJ Kashiwaya Y King MT Veech RL. A dietary ketone ester normalizes abnormal behavior in a mouse model of Alzheimer's disease. Int J Mol Sci. (2020) 21:1044. 10.3390/ijms21031044 32033248 PMC7036949

[18] Kashiwaya Y Bergman C Lee JH Wan R King MT Mughal MR. A ketone ester diet exhibits anxiolytic and cognition-sparing properties, and lessens amyloid and tau pathologies in a mouse model of Alzheimer's disease. Neurobiol Aging. (2013) 34:1530–9. 10.1016/j.neurobiolaging.2012.11.023 23276384 PMC3619192

[19] Hardy J Allsop D. Amyloid deposition as the central event in the etiology of Alzheimer's disease. Trends Pharmacol Sci. (1991) 12:383–8. 10.1016/0165-6147(91)90609-V 1763432

[20] Jawhar S Trawicka A Jenneckens C Bayer T Wirths O. Motor deficits, neuron loss, and reduced anxiety coinciding with axonal degeneration and intraneuronal abeta aggregation in the 5XFAD mouse model of Alzheimer's disease. Neurobiol Aging. (2012) 33:196.e29–40. 10.1016/j.neurobiolaging.2010.05.027 20619937

[21] Johansson M Stomrud E Lindberg O Westman E Johansson PM van Westen D. Apathy and anxiety are early markers of Alzheimer's disease. Neurobiol Aging. (2020) 85:74–82. 10.1016/j.neurobiolaging.2019.10.008 31735378

[22] Nistor M Don M Parekh M. Alpha- and beta-secretase activity as a function of age and beta-amyloid in down syndrome and normal brain. Neurobiol Aging. (2007) 28:1493–506. 10.1016/j.neurobiolaging.2006.06.023 16904243 PMC3375834

[23] Lott IT Head E. Alzheimer disease and down syndrome: factors in pathogenesis. Neurobiol Aging. (2005) 26:383–9. 10.1016/j.neurobiolaging.2004.08.005 15639317

[24] Shen ZX. Brain cholinesterases: II. The molecular and cellular basis of Alzheimer's disease. Med Hypotheses. (2004) 63:308–21. 10.1016/j.mehy.2004.02.031 15236795

[25] Wenk GL. Neuropathologic changes in Alzheimer's disease. J Clin Psychiatry. (2003) 64:7–10. 12934968

[26] Nguyen TT Ta QTH Nguyen TK O Nguyen TTD. van Giau V. Type 3 diabetes and its role implications in Alzheimer's disease. Int J Mol Sci. (2020) 21:3165. 10.3390/ijms21093165 32365816 PMC7246646

[27] Bredesen DE. Metabolic profiling distinguishes three subtypes of Alzheimer's disease. Aging. (2015) 7:595. 10.18632/aging.100801 26343025 PMC4586104

[28] Cunnane S Nugent S Roy M Courchesne-Loyer A Croteau E Tremblay S. Brain fuel metabolism, aging, and Alzheimer's disease. Nutrition. (2011) 27:3–20. 10.1016/j.nut.2010.07.021 21035308 PMC3478067

[29] Rodriguez-Vieitez E Saint-Aubert L Carter SF Almkvist O Farid K Schöll M. Diverging longitudinal changes in astrocytosis and amyloid PET in autosomal dominant Alzheimer's disease. Brain. (2016) 139(Pt 3):922–36. 10.1093/brain/awv404 26813969 PMC4766380

[30] Patrick RP. Role of phosphatidylcholine-DHA in preventing APOE4-associated Alzheimer's disease. FASEB J. (2019) 33:1554–64. 10.1096/fj.201801412R 30289748 PMC6338661

[31] Javitt DC. Glutamate as a therapeutic target in psychiatric disorders. Mol Psychiatry. (2004) 9:984–97. 10.1038/sj.mp.4001551 15278097

[32] Gazulla J Cavero-Nagore M. Glutamate and Alzheimer's disease. Rev Neurol. (2006) 42:427–32. 10.33588/rn.4207.2005223 16602060

[33] Kim SH Steele JW Lee SW Clemenson GD Carter TA Treuner K. Proneurogenic group II mGluR antagonist improves learning and reduces anxiety in Alzheimer Aβ oligomer mouse. Mol Psychiatry. (2014) 19:1235–42. 10.1038/mp.2014.87 25113378 PMC4217144

[34] Castellano C Cestari V Ciamei A. NMDA receptors and learning and memory processes. Curr Drug Targets. (2001) 2:273–83. 10.2174/1389450013348515 11554552

[35] Barkus C McHugh SB Sprengel R Seeburg PH Rawlins JNP Bannerman DM. Hippocampal NMDA receptors and anxiety: at the interface between cognition and emotion. Eur J Pharmacol. (2010) 626:49–56. 10.1016/j.ejphar.2009.10.014 19836379 PMC2824088

[36] Dang YH Ma XC Zhang JC Ren Q Wu J Gao CG. Targeting of NMDA receptors in the treatment of major depression. Curr Pharm. (2014) 20:5151–9. 10.2174/1381612819666140110120435 24410564

[37] Talantova M Sanz-Blasco S Zhang X Xia P Akhtar MW Okamoto S el al. Aβ induces astrocytic glutamate release, extrasynaptic NMDA receptor activation, and synaptic loss. Proc Natl Acad Sci U S A. (2015) 112:E3630. 10.1073/pnas.1511280112 26085137 PMC4500241

[38] Amani M Zolghadrnasab M Salari AA. NMDA receptor in the hippocampus alters neurobehavioral phenotypes through inflammatory cytokines in rats with sporadic Alzheimer-like disease. Physiol Behav. (2019) 202:52–61. 10.1016/j.physbeh.2019.01.005 30641081

[39] Penner J Rupsingh R Smith M Wells JL Borrie MJ Bartha R. Increased glutamate in the hippocampus after galantamine treatment for Alzheimer disease. Prog Neuropsychopharmacol Biol Psychiatry. (2010) 34:104–10. 10.1016/j.pnpbp.2009.10.007 19833161

[40] Sanacora G Kendell SF Levin Y Simen AA Fenton LR Coric V. Preliminary evidence of riluzole efficacy in antidepressant-treated patients with residual depressive symptoms. Biol Psychiatry. (2007) 61:822–5. 10.1016/j.biopsych.2006.08.037 17141740 PMC2754299

[41] Mathew SJ Amiel JM Coplan JD Fitterling HA Sackeim HA Gorman JM. Open-label trial of riluzole in generalized anxiety disorder. Am J Psychiatry. (2005) 162:2379–81. 10.1176/appi.ajp.162.12.2379 16330605

[42] Hardingham GE Fukunaga Y Bading H. Extrasynaptic NMDARs oppose synaptic NMDARs by triggering CREB shut-off and cell death pathways. Nat Neurosci. (2002) 5:405–14. 10.1038/nn835 11953750

[43] Hetman M Kharebava G. Survival signaling pathways activated by NMDA receptors. Curr Top Med Chem. (2006) 6:787–99. 10.2174/156802606777057553 16719817

[44] Magistretti PJ Pellerin L Rothman DL Shulman RG. Energy on demand. Science. (1999) 283:496–7. 10.1126/science.283.5401.496 9988650

[45] Attwell D Laughlin SB. An energy budget for signaling in the grey matter of the brain. J Cerb Blood Flow Metab. (2001) 21:1135–45. 10.1097/00004647-200110000-00001 11598490

[46] Wang R Reddy PH. Role of glutamate and NMDA receptors in Alzheimer's disease. J Alzheimers Dis. (2017) 57:1041–8. 10.3233/JAD-160763 27662322 PMC5791143

[47] van der Auwera I Wera S van Leuven F Henderson ST. A ketogenic diet reduces amyloid beta 40 and 42 in a mouse model of Alzheimer's disease. Nutr Metab. (2005) 2:1–8. 10.1186/1743-7075-2-28 16229744 PMC1282589

[48] Reger MA Henderson ST Hale C Cholerton B Baker LD Watson GS. Effects of β-hydroxybutyrate on cognition in memory-impaired adults. Neurobiol Aging. (2004) 25:311–4. 10.1016/S0197-4580(03)00087-3 15123336

[49] Gano LB Patel M Rho JM. Ketogenic diets, mitochondria, and neurological diseases. J Lipid Res. (2014) 55:2211–28. 10.1194/jlr.R048975 24847102 PMC4617125

[50] Fedorovich SV Voronina PP. Waseem TV. Ketogenic diet versus ketoacidosis: what determines the influence of ketone bodies on neurons?. Neural Regen Res. (2018) 13:2060. 10.4103/1673-5374.241442 30323121 PMC6199956

[51] Olson CA Vuong HE Yano JM Liang QY Nusbaum DJ Hsiao EY. The gut microbiota mediates the anti-seizure effects of the ketogenic diet. Cell. (2018) 173:1728–41. 10.1016/j.cell.2018.04.027 29804833 PMC6003870

[52] Pinto A Bonucci A Maggi E Corsi M Businaro R. Anti-oxidant and anti-inflammatory activity of ketogenic diet: new perspectives for neuroprotection in Alzheimer's disease. Antioxidants. (2018) 7:63. 10.3390/antiox7050063 29710809 PMC5981249

[53] Taylor MK Swerdlow RH Burns JM Sullivan DK. An experimental ketogenic diet for Alzheimer disease was nutritionally dense and rich in vegetables and avocado. Curr Dev Nutr. (2019) 3:nzz003. 10.1093/cdn/nzz003 30931426 PMC6435445

[54] Włodarek D. Role of ketogenic diets in neurodegenerative diseases (Alzheimer's disease and Parkinson's disease). Nutrients. (2019) 11:169. 10.3390/nu11010169 30650523 PMC6356942

[55] Brandt J Buchholz A Henry-Barron B Vizthum D Avramopoulos D Cervenka MC. preliminary report on the feasibility and efficacy of the modified atkins diet for treatment of mild cognitive impairment and early Alzheimer's disease. J Alzheimers Dis. (2019) 68:969–81. 10.3233/JAD-180995 30856112

[56] Henderson ST Vogel JL Barr LJ Garvin F Jones JJ Costantini LC. Study of the ketogenic agent AC-1202 in mild to moderate Alzheimer's disease: a randomized, double-blind, placebo-controlled, multicenter trial. Nutr Metab (Lond). (2009) 6:31. 10.1186/1743-7075-6-31 19664276 PMC2731764

[57] Yang X Cheng B. Neuroprotective and anti-inflammatory activities of ketogenic diet on MPTP-induced neurotoxicity. J Mol Neurosci. (2010) 42:145–53. 10.1007/s12031-010-9336-y 20333481

[58] de la Rubia Ortí JE García-Pardo MP Drehmer E Sancho Cantus D Julián Rochina M Aguilar MA. Improvement of main cognitive functions in patients with Alzheimer's disease after treatment with coconut oil enriched mediterranean diet: a pilot study. J Alzheimers Dis. (2018) 65:577–87. 10.3233/JAD-180184 30056419

[59] Sonnay S Chakrabarti A Thevenet J Wiederkehr A Christinat N Masoodi M. Differential metabolism of medium-chain fatty acids in differentiated human-induced pluripotent stem cell-derived astrocytes. Front Physiol. (2019) 10:657. 10.3389/fphys.2019.00657 31214043 PMC6558201

[60] Thevenet J de Marchi U Domingo JS Christinat N Bultot L Lefebvre G. Medium-chain fatty acids inhibit mitochondrial metabolism in astrocytes promoting astrocyte-neuron lactate and ketone body shuttle systems. FASEB J. (2016) 30:1913–26. 10.1096/fj.201500182 26839375

[61] Iglesias J Morales L Barreto GE. Metabolic and inflammatory adaptation of reactive astrocytes: role of PPARs. Mol Neurobiol. (2017) 54:2518–38. 10.1007/s12035-016-9833-2 26984740

[62] Pflanz NC Daszkowski AW James KA Mihic SJ. Ketone body modulation of ligand-gated ion channels. Neuropharmacology. (2019) 148:21–30. 10.1016/j.neuropharm.2018.12.013 30562540

[63] Lund TM Risa O Sonnewald U Schousboe A Waagepetersen HS. Availability of neurotransmitter glutamate is diminished when beta-hydroxybutyrate replaces glucose in cultured neurons. J Neurochem. (2009) 110:80–91. 10.1111/j.1471-4159.2009.06115.x 19457063

[64] Nafar F Clarke JP Mearow KM. Coconut oil protects cortical neurons from amyloid beta toxicity by enhancing signaling of cell survival pathways. Neurochem Int. (2017) 105:64–79. 10.1016/j.neuint.2017.01.008 28126466

[65] Pavlakis SG Phillips PC DiMauro S de Vivo DC Rowland LP. Mitochondrial myopathy, encephalopathy, lactic acidosis, and strokelike episodes: a distinctive clinical syndrome. Ann Neurol. (1984) 16:481–8. 10.1002/ana.410160409 6093682

[66] Hertz L Chen Y Waagepetersen HS. Effects of ketone bodies in Alzheimer's disease in relation to neural hypometabolism, β-amyloid toxicity, and astrocyte function. J Neurochem. (2015) 134:7–20. 10.1111/jnc.13107 25832906

[67] Farah BA. Effects of caprylic triglyceride on cognitive performance and cerebral glucose metabolism in mild Alzheimer's disease: a single-case observation. Front Aging Neurosci. (2014) 16:1–4. 10.3389/fnagi.2014.00133 25076901 PMC4099555

[68] Moretti A Gorini A Villa RF. Affective disorders, antidepressant drugs and brain metabolism. Mol Psychiatry. (2003) 8:773–85. 10.1038/sj.mp.4001353 12931205

[69] Vucic Lovrencic M Pibernik-Okanovic M Sekerija M Prasek M Ajdukovic D Kos J. Improvement in depressive symptoms is associated with reduced oxidative damage and inflammatory response in type 2 diabetic patients with subsyndromal depression: the results of a randomized controlled trial comparing psychoeducation, physical exercise, and enhanced treatment as usual. Int J Endocrinol. (2015) 2015:210406. 10.1155/2015/210406 26347775 PMC4546977

[70] Labrenz F Ferri F Wrede K Forsting M Schedlowski M Engler H. Altered temporal variance and functional connectivity of BOLD signal is associated with state anxiety during acute systemic inflammation. Neuroimage. (2019) 184:916–24. 10.1016/j.neuroimage.2018.09.056 30243957

[71] Stafstrom CE Rho JM. The ketogenic diet as a treatment paradigm for diverse neurological disorders. Front Pharmacol. (2012) 3:59. 10.3389/fphar.2012.00059 22509165 PMC3321471

[72] Cheng B Yang X An L Gao B Liu X Liu S. Ketogenic diet protects dopaminergic neurons against 6-OHDA neurotoxicity *via* up-regulating glutathione in a rat model of Parkinson's disease. Brain Res. (2009) 1286:25–31. 10.1016/j.brainres.2009.06.060 19559687

[73] Crane PK Walker R Hubbard RA Li G Nathan DM Zheng H. Glucose levels and risk of dementia. N Engl J Med. (2013) 369:540–8. 10.1056/NEJMoa1215740 23924004 PMC3955123

[74] Croteau E Castellano CA Fortier M Bocti C Fulop T Paquet N. A cross-sectional comparison of brain glucose and ketone metabolism in cognitively healthy older adults, mild cognitive impairment and early Alzheimer's disease. Exp Gerontol. (2018) 107:18–26. 10.1016/j.exger.2017.07.004 28709938

[75] Liu L Komatsu H Murray IV Axelsen PH. Promotion of amyloid beta protein misfolding and fibrillogenesis by a lipid oxidation product. J Mol Biol. (2008) 377:1236–50. 10.1016/j.jmb.2008.01.057 18304576

[76] Gottlieb RA Carreira RS. Autophagy in health and disease. 5 Mitophagy as a way of life. Am J Physiol Cell Physiol. (2010) 299:C203–10. 10.1152/ajpcell.00097.2010 20357180 PMC2928637

[77] Smith MA Harris PLR Sayre LM Perry G. Iron accumulation in Alzheimer disease is a source of redox-generated free radicals. Proc Natl Acad Sci USA. (1997) 94:9866–8. 10.1073/pnas.94.18.9866 9275217 PMC23283

[78] Veurink G Perry G Singh SK. Role of antioxidants and a nutrient rich diet in Alzheimer's disease. Open Biol. (2020) 10:200084. 10.1098/rsob.200084 32543351 PMC7333894

[79] Zandi PP Anthony JC Khachaturian AS Stone SV Gustafson D Tschanz JT. Reduced risk of Alzheimer disease in users of antioxidant vitamin supplements: the cache county study. Arch Neurol. (2004) 61:82–8. 10.1001/archneur.61.1.82 14732624

[80] Maczurek A Hager K Kenklies M Sharman M Martins R Engel J. Lipoic acid as an anti-inflammatory and neuroprotective treatment for Alzheimer's disease. Adv Drug Deliver Rev. (2008) 60:1463–70. 10.1016/j.addr.2008.04.015 18655815

[81] Menard C Bastianetto S Quirion R. Neuroprotective effects of resveratrol and epigallocatechin gallate polyphenols are mediated by the activation of protein kinase C gamma. Front Cell Neurosci. (2013) 7:281. 10.3389/fncel.2013.00281 24421757 PMC3872731

[82] Sharma K. Cholinesterase inhibitors as Alzheimer's therapeutics (review). Mol Med Report. (2019) 20:1479–87. 10.3892/mmr.2019.10374 31257471 PMC6625431

[83] Yuanyuan J Junyan Z Cuola D Jingjing C Yuhui S Dan X. Memantine attenuated alcohol withdrawal-induced anxiety-like behaviors through down-regulating NR1-CaMKII-ERK signaling pathway. Neurosci Lett. (2018) 686:133–9. 10.1016/j.neulet.2018.09.006 30213620

[84] Czarnecka K Chuchmacz J Wójtowicz P Szymański P. Memantine in neurological disorders - schizophrenia and depression. J Mol Med. (2021) 99:327–34. 10.1007/s00109-020-01982-z 33447926 PMC7900025

[85] Bowen RC Balbuena L Baetz M. Lamotrigine reduces affective instability in depressed patients with mixed mood and anxiety disorders. J Clin Psychopharmacol. (2014) 34:747–9. 10.1097/JCP.0000000000000164 24943394

[86] Herman E. Lamotrigine: a depression mood stabiliser. Eur Neuropsychopharmacol. (2004) 14 Suppl 2:S89–93. 10.1016/j.euroneuro.2004.03.003 15142613

[87] Fortier M Castellano C-A Croteau E Langlois F Bocti C St-Pierre V. A ketogenic drink improves brain energy and some measures of cognition in mild cognitive impairment. Alzheimers Dement J Alzheimers Assoc. (2019) 15:625–34. 10.1016/j.jalz.2018.12.017 31027873

[88] Taylor MK Sullivan DK Mahnken JD Burns JM Swerdlow RH. Feasibility and efficacy data from a ketogenic diet intervention in Alzheimer's disease. Alzheimers Dement. (2018) 4:28–36. 10.1016/j.trci.2017.11.002 29955649 PMC6021549

[89] Krikorian R Shidler MD Dangelo K Couch SC Benoit SC Clegg DJ. Dietary ketosis enhances memory in mild cognitive impairment. Neurobiol Aging. (2012) 33:425. 10.1016/j.neurobiolaging.2010.10.006 21130529 PMC3116949

[90] McDonald TJW Cervenka MC. Ketogenic diets for adult neurological disorders. Neurotherapeutics. (2018) 15:1018–31. 10.1007/s13311-018-0666-8 30225789 PMC6277302

[91] Klein P Janousek J Barber A Weissberger R. Ketogenic diet treatment in adults with refractory epilepsy. Epilepsy Behav. (2010) 19:575–9. 10.1016/j.yebeh.2010.09.016 20937568

[92] Lilamand M Porte B Cognat E Hugon J Mouton-Liger F Paquet C. Are ketogenic diets promising for Alzheimer's disease? A translational review. Alzheimers Res Ther. (2020) 12:42. 10.1186/s13195-020-00615-4 32290868 PMC7158135

[93] Ting R Dugré N Allan GM Lindblad AJ. Ketogenic diet for weight loss. Can Fam Physician. (2018) 64:906. 30541806 PMC6371871

[94] Cova I Clerici F Rossi A Cucumo V Ghiretti R Maggiore L. Weight loss predicts progression of mild cognitive impairment to Alzheimer's disease. PLoS ONE. (2016) 11:e0151710. 10.1371/journal.pone.0151710 26990757 PMC4798596

